# Vasopressin Inhibits LTP in the CA2 Mouse Hippocampal Area

**DOI:** 10.1371/journal.pone.0049708

**Published:** 2012-12-07

**Authors:** Magda Chafai, Maithé Corbani, Gilles Guillon, Michel G. Desarménien

**Affiliations:** 1 CNRS, UMR-5203, Institut de Génomique Fonctionnelle, Montpellier, France; 2 INSERM, U661, Montpellier, France; 3 Universités de Montpellier 1 & 2, UMR-5203, Montpellier, France; Centre national de la recherche scientifique, University of Bordeaux, France

## Abstract

Growing evidence points to vasopressin (AVP) as a social behavior regulator modulating various memory processes and involved in pathologies such as mood disorders, anxiety and depression. Accordingly, AVP antagonists are actually envisaged as putative treatments. However, the underlying mechanisms are poorly characterized, in particular the influence of AVP on cellular or synaptic activities in limbic brain areas involved in social behavior. In the present study, we investigated AVP action on the synapse between the entorhinal cortex and CA2 hippocampal pyramidal neurons, by using both field potential and whole-cell recordings in mice brain acute slices. Short application (1 min) of AVP transiently reduced the synaptic response, only following induction of long-term potentiation (LTP) by high frequency stimulation (HFS) of afferent fibers. The basal synaptic response, measured in the absence of HFS, was not affected. The Schaffer collateral-CA1 synapse was not affected by AVP, even after LTP, while the Schaffer collateral-CA2 synapse was inhibited. Although investigated only recently, this CA2 hippocampal area appears to have a distinctive circuitry and a peculiar role in controlling episodic memory. Accordingly, AVP action on LTP-increased synaptic responses in this limbic structure may contribute to the role of this neuropeptide in controlling memory and social behavior.

## Introduction

The influence of vasopressin (AVP) in memory was suggested 30 years ago [1] but it is only recently that appropriate pharmacological tools have provided convincing evidence concerning its involvement in memory and in social and stress-related behaviors [2], [3], [4], [5], [6], [7]. Vasopressinergic neurons with central projections are mainly located in the paraventricular nucleus, suprachiasmatic nucleus, bed nucleus of the stria terminalis and amygdala. They project to various structures including septum, amygdala, habenula and hippocampus (see [8], [9] where the appropriate V1 receptors (V1a and/or V1b) are expressed [10], [11], [12], [13], [14], [15]. Rats selected for high anxiety exhibit overexpression of AVP [16] and administration of AVP receptors antagonists decreases anxiety and depressive-like behaviors in rodents (see [4], [17], [18]. V1a or V1b receptors knock-out mice have been reported to display an altered anxiety behavior [4], [19] but this trait is not always reproduced in other studies and the main consequences of these invalidations are differential modifications in aggression behaviour and memory : decreased spatial memory in V1a KO mice, impaired social and episodic memories in V1b KO mice (see [4], [20], [21], [22], [23], [24]). The fact that V1a and V1b receptors knock-out mice show deficits in memory points toward central actions of AVP and involvement of hippocampal structures. Altogether, these data indicate that many affective disorders are related to excessive AVP function. However, the cellular mechanisms by which this neuropeptide acts on brain structures have not been thoroughly investigated.

Although few vasopressinergic fibers innervate the dorsal hippocampus [8], [25], both V1a and V1b receptors are abundant in this structure [14], [15]. The CA2 hippocampal subfield is particularly relevant to study central AVP actions. Indeed, although the V1a receptor is mainly distributed in the dentate gyrus and on dispersed cells in the three CA areas of the dorsal and ventral hippocampus [15], the V1b receptor is mainly expressed in the dorsal CA2 area [14]. Accordingly, implication of the CA2 area in the AVP actions on social behavior and episodic memory has been suggested [14], [20]. CA2 is not simply a transition area between CA1 and CA3 but also possesses a number of specific characteristics. Functionally, it is resistant to temporal epilepsy [26] and might be involved in the onset of schizophrenia [27]. Moreover, this area displays unique anatomical (neuronal morphology and expression of specific proteins) and electrophysiological features indicating that it is engaged differently by synaptic inputs as compared to CA3 or CA1 (see [28]). Furthermore, CA2 constitutes the key structure of a disynaptic cortico-hippocampal loop [29], [30], in which the CA2 pyramidal cells are directly activated by cortical afferents, bypassing the dentate gyrus and CA3 intermediates. The presence of specific synaptic circuitry in CA2 is also indicated by the distinct morphology and electrophysiological characteristics of parvalbumin immunoreactive interneurons [31].

In the present study, we demonstrate that AVP modulates the disynaptic cortico-hippocampal loop by inhibiting the entorhinal cortex-CA2 pyramidal neurons excitatory synapse during long-term potentiation (LTP).

## Materials and Methods

### Hippocampal slice preparation

All experiments were carried out in accordance with French/European guidelines (Agreement n° 34.128). C57BL6 male mice (4–6 weeks old) were anesthetized by isoflurane inhalation and decapitated. Brain was rapidly removed and sagittal slices (300–350 µm thick) containing the dorsal hippocampus were cut on a vibratome (Integraslice 7550, Campden Inst.,UK) in ice-cold solution containing (in mM: 195 sucrose, 10 NaCl, 2.5 KCl, 1.25 NaH_2_PO_4_, 26 NaHCO_3_, 15 glucose, 1 CaCl_2_ and 2 MgCl_2_). Freshly cut slices were then transferred to artificial cerebral spinal fluid (ACSF ; in mM: 110 NaCl, 1.2 KCl, 1.2 KH_2_PO_4_, 26 NaHCO_3_, 10 glucose, 2 CaCl_2_ and 2 MgCl_2_) at 32°C for 20 min and then at room temperature for at least 1.5 hour before recording. All solutions were saturated with 95% O_2_ and 5% CO_2_ (pH 7.4).

### Anatomical identification of CA2 pyramidal neurons

**Figure 1:**
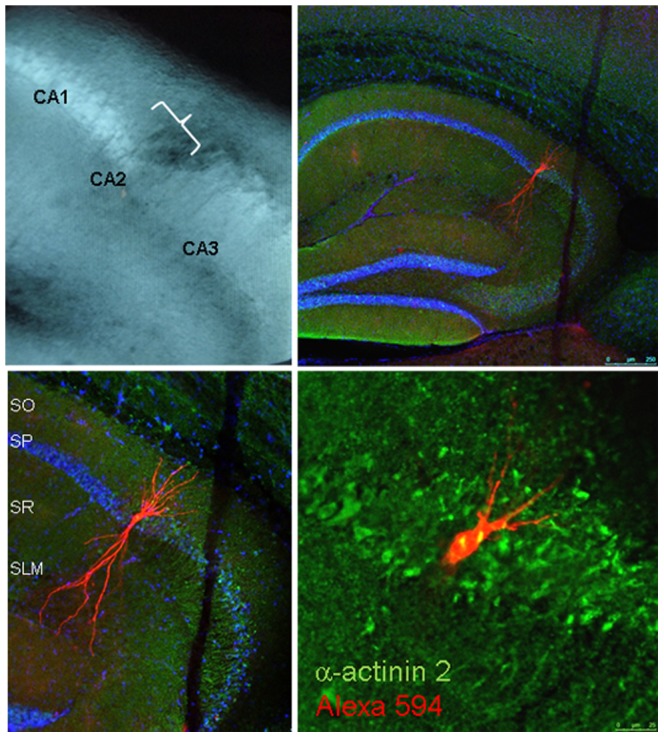
Recorded neurons are localized in the CA2 area and display a typical dendritic morphology. Upper left: Bright field image of the hippocampal slice as seen during the experiment. Note that the CA2 is well delimited between the large pyramidal layer and mossy fiber tract of the CA3 area on the right and the thin pyramidal layer of the CA1 area on the left. Upper right: Fluorescence image (×5) of a neuron filled with Alexa-594-cadaverine (Red) during whole-cell recording and typically located in the CA2 area. Blue: Hoechst labeling of cell nuclei. Lower left: Higher magnification (×10) confirms that the injected neuron in located in the characteristic dilatation of the pyramidal layer in the CA2 area and displays the typical morphological characteristics of a pyramidal CA2 neuron, with few dendritic branches in stratum oriens (SO) and a dense branching in stratum lacunosum molecular (SLM). Lower right: Merged high magnification (×40) fluorescence image showing in red the Alexa-594-cadaverine filling the injected neuron and in green the immunolabeling of α-actinin2, a protein enriched in CA2 neurons. Note the co-localization of cadaverin and α-actinin, demonstrating that the recorded neuron was located in the CA2 area. SO, stratum oriens; SP, stratum pyramidale; SR, stratum radiatum; SLM, stratum lacunosum moleculare.

In the whole-cell recording configuration, some cells were injected with Alexa fluor 594 cadaverin dye (Sigma, 50 µM in the patch pipette) and the slice was fixed immediately after recording in paraformaldehyde (4% in phosphate buffer). The slice was then processed for immunohistochemistry using a rabbit anti-α-actinin2 primary antibody (Sigma, 1/50, 48 h incubation at 4°C) and a donkey anti-rabbit CY5 secondary antibody (1/1200, 3 h at room temperature). Slices were then mounted with mowiol and imaged by confocal fluorescence microscopy (Fig. 1). The typical anatomy of the apical dendrite arborisation, with very few proximal oblique dendritic branches and a dense distal branching in the *stratum lacunosum moleculare*, together with the colocalization with α-actinin2 immunoreactivity, ascertain the fact that we recorded from pyramidal neurons of the CA2 area (see [29]). Further confirmation was obtained by using electrophysiological characteristics of CA2 pyramidal neurons (see next).

### Electrophysiological recordings

Slices were transferred to a recording chamber and continuously perfused with ACSF (32°C). Recordings were made in the dorsal hippocampus, from the soma or dendrites of CA2 neurones in the pyramidal cell layer (Fig. 1) located in the area following the end of the large mossy fibre track connecting the dentate gyrus to CA3 [29]. Stimulation (10–30 V; 0.02–0.2 ms; 0.2 Hz) of the Schaffer collaterals (SC) in the *stratum radiatum* or of the entorhinal fibers (EC LIII) in the middle of the *stratum lacunosum moleculare* was performed using a monopolar pipette filled with ACSF or a bipolar tungsten electrode. LTP was induced by high frequency stimulation (HFS: 100 pulses at 100 Hz, repeated twice at 20 s intervals) after 5–10 min baseline recording. Extracellular field potentials were recorded with a patch pipette (3–4 MΩ) containing ACSF. Whole-cell recordings were accomplished under visual control with a patch pipette (4–5 MΩ) containing (in mM): 130 KMeSO_3_, 9 KCl; 8 NaCl, 0.1 EGTA-Na, 10 HEPES-NaOH, 2 pyruvate, 2 malate, 0.5 NH_2_PO_4_; 0.5 cAMP, 2 ATP-Mg, 0.5 GTP-Tris, 14 phosphocreatine, 0.1 leupeptine, 1 MgCl_2_ (pH 7.2, 295–300 mOsm). In order to further ascertain that recordings were indeed performed from CA2 pyramidal cells, action potentials were evoked by a sustained depolarization (30 ms) and we controlled for the presence of a delay before the first spike, as well as for the absence of a slow post-spike hyperpolarizing potential (see [29]). Recordings were performed with an axopatch 2B amplifier (Axon instruments, DIPSI, Paris). Data were acquired, stored and analysed using the PClamp software (Axon Instrument, DIPSI, Paris). Arg^8^AVP (10–100 nM; Bachem, Switzerland) was bath-applied during 1 min, before or 30–40 min after HFS. All reagents were purchased from Sigma (L'isle d'Abeau Chesnes, France) except isoflurane (Nicholas Piramal Limited, London, UK). Numerical data are expressed as mean ± SEM. Differences between groups were assessed by using the Kruskal-Wallis test. Differences with *p*<0.05 were considered significant.

## Results

### Effect of AVP on individual pyramidal CA2 and CA1 cells

**Figure 2:**
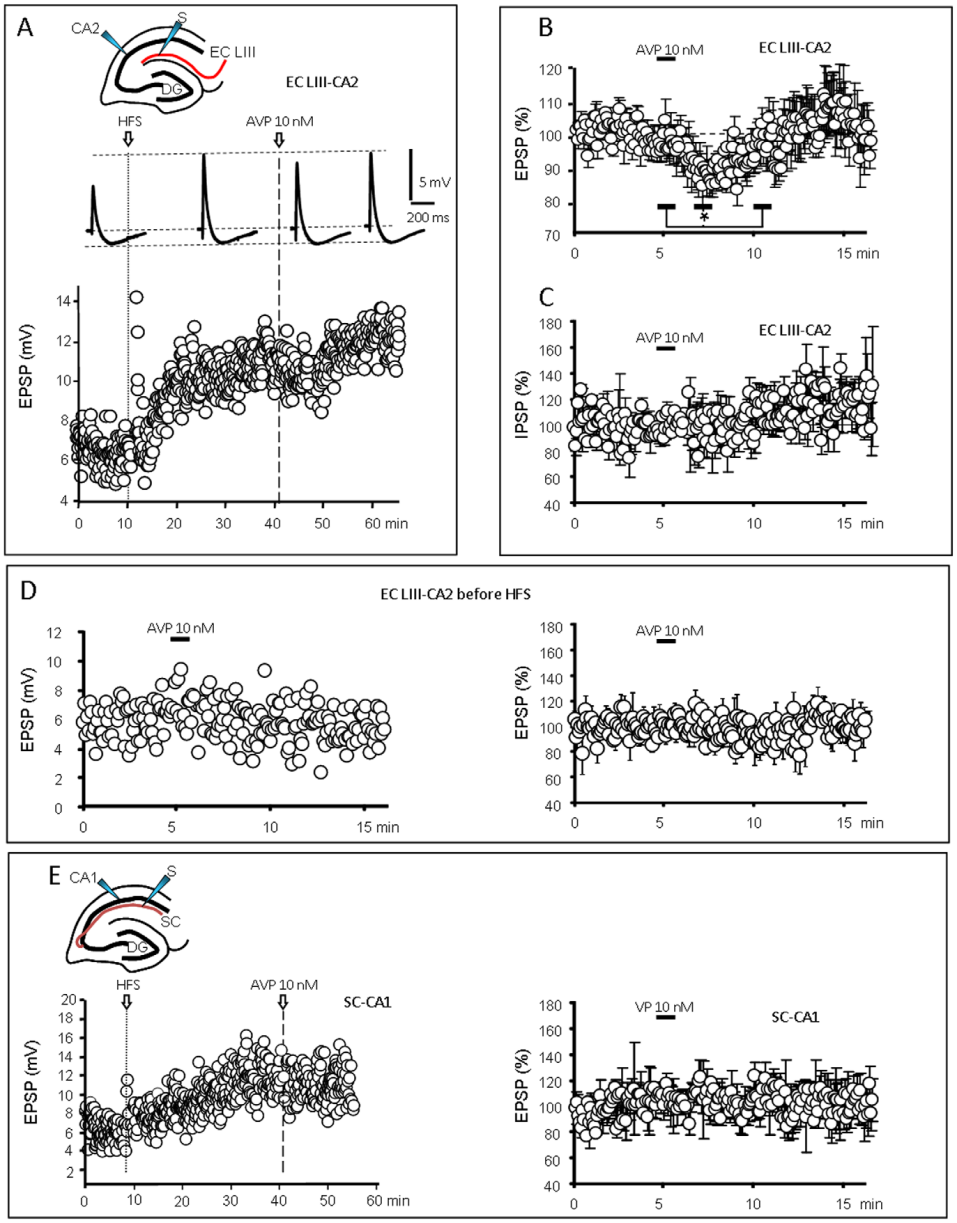
Vasopressin decreases EPSPs in CA2 after LTP induction. A: Top: typical recordings of the combined EPSP/IPSP evoked by stimulation of LIII entorhinal fibers (EC LIII) and recorded in CA2 pyramidal neurons (inset). Traces are averages of 12 successive responses recorded during 1 min, before high frequency stimulation (HFS) and during LTP before, during and after the response to AVP (10 nM ; 1 min). Bottom graph: typical example showing that the amplitude of the EPSP increased transiently (short-term facilitation) after HFS and then progressively to reach a plateau (long-term potentiation: LTP). Vasopressin (AVP 10 nM; 1 min) applied during LTP transiently decreased the EPSP amplitude. B) Average graph of EPSP amplitude *vs.* time (n = 6; * *p*<0.05) demonstrating that AVP inhibited the CA2 EPSP evoked by EC LIII stimulation during LTP. Values are expressed as % of the mean of the responses recorded during 5 min before AVP application. C) Average graph (n = 6) showing that the IPSP component of the CA2 response to EC LIII stimulation was not affected by AVP. D) Typical graph (Left) and average graph (Right, n = 10) of EPSP amplitude *vs.* time showing that AVP did not affect the basal EC LIII-CA2 EPSP recorded before HFS stimulation. E) Left, Typical graph of the fEPSP evoked by Schaffer collaterals stimulation and recorded in a CA1 pyramidal neuron (SC-CA1); AVP did not affect the amplitude of the LTP-potentiated EPSP , as shown on the average graph (Right, n = 4).

Pyramidal cells from the dorsal CA2 area were recorded using the whole-cell configuration of the patch-clamp technique. To avoid perturbation of synaptic potentials by spontaneous action potentials, the membrane potential was maintained close to −80 mV. Somatic EPSPs were evoked in CA2 neurons in response to stimulation of EC LIII inputs and their amplitude was measured in the absence or presence of 10 nM AVP, before and after HFS-induced LTP (Fig. 2A). CA2 neurons exhibited combined EPSPs and IPSPs (Fig. 2A) and HSF induced a robust LTP of the EPSP only (to 180±17% of control values before HFS; Fig. 2A) in 14/21 neurons. Bath application of AVP (10 nM; 1 min) during efficient LTP (178±31% of control; n = 6) transiently reduced the EPSP amplitude by 16.8±2.2% (n = 6, Fig. 2A, 2B; *p*<0.05 when comparing the EPSPs amplitude for 1 min during the AVP application, at peak of the response 3 min latter and after returning to control values 6 min after AVP application), whereas the amplitude of the IPSP remained unchanged (Fig. 2C; n = 6). AVP had no effect on the synaptic response when applied in the absence of HFS (n = 10; Fig. 2D). Furthermore, in neurons in which HSF did not induce LTP (EPSP amplitude = 99±10% of control; n = 7), no modification in the synaptic response was observed after AVP application (n = 7; not shown). To test whether this effect of AVP on CA2 pyramidal neurons was generalized to hippocampal pyramidal cells or not, we investigated its action on the classically studied SC-CA1 synapse after HFS-induced LTP. Recordings illustrated in Fig. 2E show that AVP had no effect on the amplitude of this EPSP (n = 4). In all the recordings performed, CA1 and CA2 neurons exhibited no change in resting potential (or of the current injected to maintain it close to −80 mV) related to AVP application. In conclusion, AVP induced a transient decrease of the EC LIII-CA2 EPSP only during LTP but affected neither the associated IPSP nor the SC-CA1 EPSP.

### Effect of AVP on the synaptic field response

**Figure 3:**
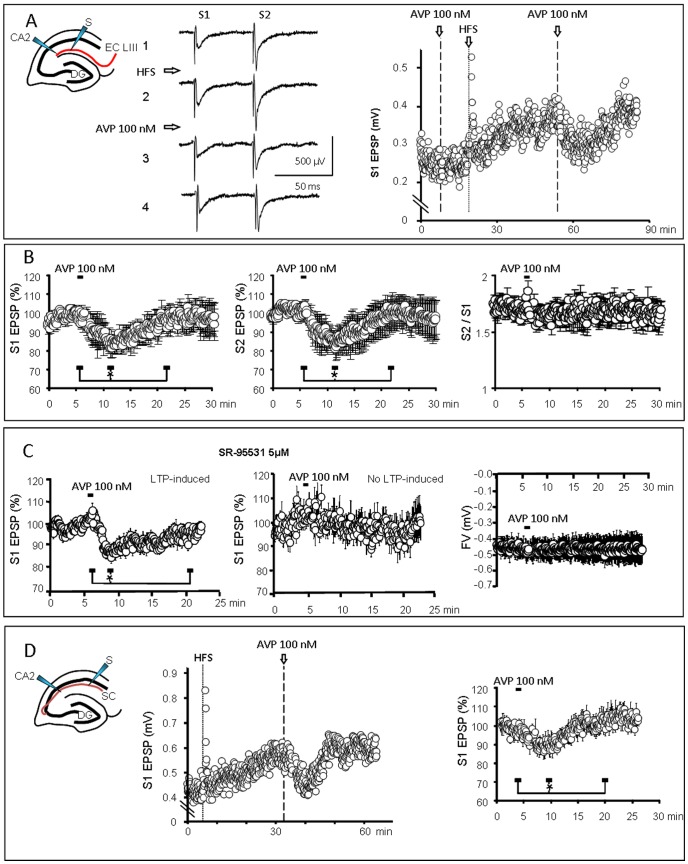
Vasopressin decreased the population fEPSP evoked by stimulation of LIII entorhinal fibers (EC LIII) and recorded in the CA2 dendritic field. A) Left : Typical recordings (left, 12 traces averaged over 1 min) of fEPSPs evoked by two successive stimulations before HFS (1) and during LTP before (2), during (3) and after (4) the response to AVP (100 nM ; 1 min). Note that the response to the second stimulation (S2) is larger than the first one (S1), indicative of a paired-pulse facilitation. Right, corresponding graph of the fEPSP amplitude *vs.* time. HFS induced a short- and then a long-term potentiation. Values correspond to S1. AVP (1 min) transiently decreased the LTP-potentiated fEPSP. B) Average graphs (n = 8; values expressed as % of the mean of the responses recorded during 5 min before AVP application) demonstrating that AVP (100 nM; 1 min) transiently and significantly decreased the amplitude of both S1 and S2 fEPSPs without affecting the paired-pulse facilitation (calculated as the ratio S2/S1). C) Left: fEPSP recorded in the presence of the GABA_A_ antagonist SR 95531. AVP was effective to decrease the fEPSP during LTP (n = 14) but failed to affect the fEPSP in slices in which no LTP was evoked (Middle; n = 7). Right, Absence of effect of AVP on the fiber volley (FV) amplitude (n = 5), demonstrating that AVP did not reduce the excitability of the EC LIII fibers. D) fEPSP recorded in the CA2 dendritic area during stimulation of SC fibers. Left, Typical recording in a slice in which HFS induced a LTP and AVP reduced the potentiated fEPSP. Right, Average graph (n = 7) demonstrating that AVP (100 nM; 1 min) transiently and significantly decreased the amplitude of the SC-CA2 fEPSPs.

In order to confirm and extend results obtained from single CA2 neurons, we investigated the response of CA2 pyramidal population to AVP application. Extracellular recordings of CA2 field EPSPs (fEPSPs) in the apical dendritic area, in response to stimulation of EC LIII inputs, validated previous whole-cell findings *i.e.* CA2 cells displayed a large LTP after HSF (154±12% n = 12; Fig. 3A). In addition, application of two successive stimulations separated by 50 ms revealed the presence of a paired-pulse facilitation (Fig. 3 A). Bath application of AVP 10 nM (1 min) had a slight but not significant effect on these successive synaptic responses (n = 10; not shown). This could result from the fact that field recordings were performed deeper in the slice than whole-cell recordings. Accordingly, we increased the AVP concentration to 100 nM, a concentration that does not activate OT receptors in similar conditions in slices from the supraoptic nucleus [32]. In this condition, bath-applied AVP (1 min) induced a significant transient diminution of both synaptic responses (S1 : −13.3±2.9% n = 8 and S2 : −14.9±5.5% n = 8, Fig. 3B; *p*<0.05 when comparing the EPSPs amplitude for 1 min during the AVP application, at peak of the response 6 min latter and after returning to control values 15 min after the application) in slices displaying a large LTP (168±16%). However, the ratio of the second over the first fEPSP was not affected by AVP (Fig. 3B). In 4 slices with a slight LTP (125±3%), AVP failed to affect the fEPSP (not shown).

To confirm data obtained in the whole-cell recording configuration and ascertain that AVP acts on the EPSP specifically, we applied it in the presence of the GABA_A_ antagonist SR 95531 (5 µM) [33]. In 14 slices in which a significant LTP was established after HFS, we observed a transient reduction of the fEPSP amplitude (Fig. 3C; S1 : −14.7±2.3% n = 14 and S2 : −12.1±2.2% n = 14, *p*<0.01 when comparing the fEPSPs amplitude for 1 min during the AVP application, at peak of the response 3 min latter and after returning to control values 15 min after AVP application), similar to that observed in control conditions. In slices in which HFS did not induce a LTP (n = 7), AVP had no effect on the fEPSP in the presence of SR 95531 (Fig. 3C).

In order to test whether AVP acts on a synaptic target or affects the excitability of afferent axons, we measured the amplitude of the fibers volley, a transient spike preceding the fEPSP that reflects the action potentials transiting in afferent axons. This spike was not affected by AVP (n = 5; Fig. 3 C).

These data confirm that AVP decreases the EC LIII-CA2 EPSP during LTP. Furthermore, the absence of effect on the fibers volley and on the paired-pulse facilitation is in favor of a postsynaptic site of action on the dendrites of CA2 pyramidal neurons. The question then arises whether AVP inhibits specifically the cortical input on CA2 neurons (the EC LIII-CA2 synapse) or more generally excitatory afferents on CA2 pyramidal neurons. Accordingly, we tested the effect of AVP (100 nM) on the SC-CA2 synapse. HFS induced a small but significant LTP (123±5%; n = 7) during which AVP reduced the amplitude of the fEPSPs (Fig. 3 D left), although to a smaller extend than for the EC LIII-CA2 synapse (S1 : −5.7±0.8% n = 7 and S2 : −6.3±1.2% n = 7, Fig. 3D right; *p*<0.05 when comparing the fEPSPs amplitude for 1 min during the AVP application, at peak of the response 6 min latter and after returning to control values 15 min after the application). In summary, these data demonstrate that AVP inhibits excitatory synapses impinging on CA2 pyramidal neurons, from the entorhinal cortex or from SC, but not the classically studied SC-CA1 synapse.

## Discussion

In the present study, using both whole-cell recordings from individual neurons and field potential recordings of population synaptic events, we demonstrate for the first time that AVP inhibits the excitatory inputs that target CA2 pyramidal neurons, including the recently described [29], [30] disynaptic entorhinal cortex-CA2-CA1 pathway. By contrast, AVP does not affect the SC-CA1 synapse. Remarkably, this inhibitory effect of AVP on the entorhinal cortex-CA2 synapse only concerns LTP-potentiated EPSPs. Considering the proposed involvement of the CA2 area in generating the theta rhythm and the influence of this rhythm on learning capabilities (see [28]), AVP-induced inhibition of LTP in CA2 neurons is a putative cellular mechanisms supporting the role of this neuropeptide in episodic memory [20].

The effect of AVP on synaptic events in the hippocampus has not been thoroughly investigated. In the rat dentate gyrus *in vitro*, Chen *et al.* [34] observed that nanomolar concentrations of AVP, *via* activation of a V1 receptor, induced a prolonged increase in slope and amplitude of the EPSP in the presence of 1.5 mM calcium and a prolonged depression in the presence of 2.5 mM calcium, but this was not reproduced *in vivo* [35]. In the guinea pig CA1 and CA3 hippocampal areas, AVP prevents LTP without affecting the basal EPSP [36]. By contrast, we observed an absence of AVP action on the mouse SC-CA1 neuron synapse. This opposite result may be explained either by a species difference or by the fact that Sakurai *et al*. (1998) applied AVP before and during the onset of LTP while we have tested AVP on an established LTP. In other central structures like motor areas [37], supraoptic nucleus [32], [38] or amygdala [39], AVP either increased or decreased excitability (see [37]). When characterized, increased excitability resulted from activation of a TTX-resistant sodium current [37]. If present in CA2 pyramidal cells, this AVP-induced current is likely either small or supported by channels distant from the soma since we did not observe any change in resting potential during whole-cell recordings of synaptic events in CA2 pyramidal neurons.

Considering the pre- or post-synaptic site of action of AVP, several arguments are in favor of a postsynaptic localization. First, the fact that AVP decreases both SC- and EC LIII- evoked EPSPs in the CA2 area, and not SC-evoked EPSPs in the CA1 area, is indicative of a location of AVP receptors on CA2 pyramidal neurons rather than on the afferent fibers. Second, we showed that AVP did not affect the excitability of the afferent fibers, as ascertained by the absence of modification of the fiber volley during AVP-evoked fEPSP decrease. Third, the absence of AVP-induced change in paired-pulse facilitation is in favor of a postsynaptic action. Indeed, this facilitation is modified in the case of presynaptic AVP actions in the supraoptic nucleus (see [40]). A post-synaptic action of AVP is also in agreement with the fact that AVP decreased only EPSPs and not IPSPs in CA2 neurons. A presynaptic action on the synaptic buttons of entorhinal cortex afferences would affect both responses. This differential effect on EPSPs also indicates a localized (synaptic?) dendritic site of action. Indeed, a general depolarization or resistive shunt of the pyramidal neuron's membrane would have affected the EPSP and IPSP equally. Finally, the hypothesis of a post-synaptic target for AVP is reinforced by the reported high density of V1b receptors, detected by immunohistology [13] or *in situ* hybridization [14] in the pyramidal layer of CA2. However, *in situ* hybridization allows identification of the cells expressing the receptor mRNA but not the localization of the protein. The precise distribution of AVP receptors in the mouse hippocampus will be facilitated by the use of new tools such as specific fluorescently labeled ligands [41], [42].

The CA2 hippocampal area, long considered as a transition zone between CA1 and CA3, is in the scope of several recent studies that demonstrate both functional and morphological distinctive characteristics [29], [30], [31]. Particularly relevant to the present study, the responses of CA2 and CA1 pyramidal cells to afferent inputs are strikingly different [28], [29]. Indeed, SC fibers stimulation evokes a large EPSP enhanced by LTP in CA1 and a smaller one with a weaker LTP in CA2. EC LIII and EC LII entorhinal fibers converge on the distant dendritic region of CA2, where they induce a large EPSP strongly increased during LTP. By contrast, EC LII-CA3 and EC LIII-CA1 synapses evoke small EPSPs. Our data indicate that these synapses do not differ only by their strength and sensitivity to LTP but also by their pharmacological properties since AVP decreases the SC-CA2 and EC LIII-CA2 synapses, an effect which is not observed in the SC-CA1 one. It will be interesting to study the effect of AVP on other inputs to pyramidal neurons in the hippocampus, in particular the EC LIII-CA1 synapses. Differential connections, sensitivity to LTP and to AVP indicate that the CA2 area is a specific site of integration of some cortical inputs, with functional implications that remain to be extensively studied. In parallel with findings showing the contribution of CA2 to learning and social behavior, it will be worth determining the pharmacological sensitivity of this area and particularly the respective contribution of the V1a and V1b receptors to AVP responses. Indeed, these receptors seem to have a different distribution in this area, as compared to the rest of the hippocampus [14].

In conclusion, our data demonstrate that AVP inhibits LTP at the hippocampal target of EC LIII and SC fibers in the CA2 area. Considering the suggested contribution of CA2 to episodic memory, this cellular effect of AVP may contribute to the central influence of this neuropeptide in memory and mood disorders.
